# Impact of a unit-wide feeding tolerance management protocol on enteral feeding outcomes in infants with congenital heart disease: a pre–post quality improvement cohort study

**DOI:** 10.3389/fresc.2026.1765642

**Published:** 2026-03-27

**Authors:** Yaqin Xiao, Yanhua Wang, Jingpu Gao, Hongxia Gong, Wenting Chen, Xiaobo Cao

**Affiliations:** 1 Department of Cardiac Surgery, Hebei Children's Hospital, Hebei Clinical Medicine Research Center for Children's Health and Diseases, Shijiazhuang, China; 2 Department of Nursing Management, Hebei Children's Hospital, Hebei Clinical Medicine Research Center for Children's Health and Diseases, Shijiazhuang, China; 3 Emergency Department, Hebei Children's Hospital, Hebei Clinical Medicine Research Center for Children's Health and Diseases, Shijiazhuang, China

**Keywords:** congenital heart disease, enteral feeding, feeding intolerance, pediatric ICU, pre–post cohort, quality improvement

## Abstract

**Background:**

Infants recovering from congenital heart disease (CHD) surgery frequently experience feeding intolerance (FI), a major barrier to achieving adequate enteral nutrition (EN). This pre–post quality improvement study evaluated whether implementation of a standardized feeding-tolerance management pathway was associated with improved consistency of EN monitoring and corresponding trends in FI-related outcomes.

**Methods:**

A single-center, consecutively enrolled quality improvement cohort included postoperative CHD infants admitted in 2022 (pre-implementation) and 2023 (post-implementation). The intervention consisted of unit-wide standardization of FI risk assessment, EN monitoring indicators, alert-trigger criteria, and documentation procedures. No feeding strategy was altered. The primary outcome was FI incidence; secondary outcomes included time to full EN, gastrointestinal symptoms, EN interruptions, and nutritional status. Statistical analyses included *χ*^2^ tests, logistic regression, Cox models, and negative binomial regression.

**Results:**

A total of 301 infants were analyzed (148 pre-implementation; 153 post-implementation). Documentation completeness increased from 68.4% to 91.7%, and adherence to predefined alert criteria rose from 54.7% to 92.2% (both *P* < 0.001). FI incidence decreased from 42.6% to 24.2% (RR 0.57, 95% CI 0.41–0.79), and adjusted odds of FI remained lower post-implementation (aOR 0.48, 95% CI 0.29–0.79). Time to full EN was shorter (median 9 to 6 days; HR 1.48, 95% CI 1.11–1.97). Gastrointestinal symptom burden and GI-related feeding interruptions were reduced, while nutritional decline was attenuated, reflected by improved weight-for-age z score (WAZ) change (ΔWAZ −0.22 to −0.04; *P* = 0.004). Subgroup and sensitivity analyses demonstrated consistent directional trends.

**Conclusions:**

Implementation of a standardized feeding-tolerance pathway was associated with improved monitoring consistency and favorable trends in FI incidence, EN progression, and in-hospital nutritional stability among postoperative CHD infants.

## Background

Infants recovering from congenital heart disease (CHD) surgery, particularly those with higher surgical complexity such as STAT categories 4–5 or single-ventricle physiology, face substantial vulnerability related to impaired perfusion, low cardiac output, and increased fragility of the intestinal barrier (1, 2). Enteral nutrition (EN) plays a central role in postoperative care in this population, supporting mucosal integrity, reducing infectious risk, and contributing to overall recovery (3–5). Current guidelines and expert consensus emphasize the importance of structured monitoring of EN tolerance to ensure safe and progressive feeding advancement, highlighting that reliable assessment often carries greater clinical relevance than simply accelerating the initiation or rate of feeding (6–8).

Feeding intolerance (FI) is reported in approximately 30%–45% of infants following congenital heart disease surgery, representing a frequent barrier to advancing enteral nutrition (9–11). Clinical manifestations such as abdominal distension, recurrent emesis, and elevated gastric residuals often necessitate temporary interruptions of feeding (12, 13). When FI persists, the cumulative impact can be substantial, including heightened risk of in-hospital nutritional deterioration reflected by declines in weight-for-age z-scores, prolonged stays in intensive care, and delays in respiratory recovery (14–16).

Emerging evidence suggests that the risk of feeding intolerance is shaped less by the specific feeding strategy and more by variability in how postoperative monitoring is performed (1, 17). Multicenter evaluations have highlighted frequent inconsistencies in documentation, such as incomplete abdominal girth measurements, irregular assessment of gastric residuals, and errors in recording FI-related symptoms, which collectively hinder timely recognition of intolerance (10, 18, 19). In pediatric cardiac intensive care, however, adherence to unified feeding-monitoring protocols remains limited. Against this backdrop, quality improvement efforts aimed at strengthening process consistency may enhance the accuracy of FI identification and support more stable enteral feeding progression in infants recovering from congenital heart surgery.

Therefore, the present study sought to examine whether implementing a standardized feeding-tolerance management pathway improved the consistency of perioperative enteral nutrition documentation and the reliability of FI monitoring. The intention was to understand how greater procedural uniformity might influence the detection of FI and the pace at which enteral nutrition can be advanced in infants recovering from congenital heart surgery.

## Methods

### Study design

This investigation was conducted as a single-center, consecutively enrolled, pre–post quality improvement study designed to evaluate the consistency of feeding-tolerance monitoring practices. The study did not involve randomization or assignment to interventions and therefore does not represent a clinical trial. All data were derived from routine clinical care within the Cardiac Intensive Care Unit of Hebei Children's Hospital, which also serves as the Hebei Clinical Medicine Research Center for Children's Health and Diseases. Two time-defined cohorts were established based on the implementation timeline of the standardized feeding-tolerance management pathway: a pre-implementation period (January–December 2022) and a post-implementation period (January–December 2023).

Infants were enrolled consecutively during the study periods without selective sampling. After application of predefined exclusion criteria, a total of 301 infants were included in the final analytic cohort, comprising 148 infants in the pre-implementation group and 153 in the post-implementation group.

The project focused on pathway consistency and process standardization as part of routine nursing quality improvement. No investigational intervention was introduced, and the study falls outside the scope of the WHO definition of a clinical trial. All data were obtained from standard clinical records without altering patient management. The study protocol was reviewed and approved by the institutional ethics committee, and informed consent was obtained in accordance with hospital requirements. Written informed consent was obtained from all participants (or from parents/legally authorized guardians in case of minors) prior to enrollment in the study.

### Inclusion criteria and exclusion criteria

Infants were eligible for inclusion if they met the following conditions:

1. A diagnosis of congenital heart disease (CHD) and a postoperative plan to receive enteral nutrition.
2. Gestational age of 37 weeks or greater at birth.
3. Admission to the Cardiac Intensive Care Unit after surgery with complete documentation of enteral feeding records.

Infants were excluded if any of the following applied:

1. Presence of major gastrointestinal malformations prior to surgery.
2. A documented history of necrotizing enterocolitis or other severe gastrointestinal disease before surgery.
3. Death or withdrawal of care within 48 h postoperatively.
4. Incomplete enteral nutrition documentation, including missing records of abdominal girth, gastric residuals, emesis, or abdominal distension.
5. Requirement for gastrointestinal tube placement for palliative decompression or other non-nutritional indications.

### Pre-implementation nursing evaluation and baseline monitoring practice

Prior to pathway implementation, feeding-tolerance monitoring was performed as part of routine CICU nursing care, including documentation of abdominal girth, gastric residuals, emesis, stool output, and abdominal distension. However, the frequency of assessments and the thresholds used to trigger escalation were not fully standardized across nurses and shifts, resulting in variability in documentation completeness and in the interpretation of potential intolerance events. As part of the unit's quality-improvement preparation, the Nursing Department conducted a brief baseline review of routine charts and bedside practice to identify the most common sources of variability (e.g., inconsistent recording frequency, non-uniform symptom terminology, and unclear escalation thresholds). These observations informed the selection of the standardized data fields, monitoring frequencies, and alert-trigger criteria incorporated into the pathway.

### Care pathway structure and clinical execution

The standardized feeding-tolerance management pathway was issued institution-wide by the Nursing Department and implemented uniformly across the Cardiac Intensive Care Unit. The pathway was developed independently of the research team, and no randomization, allocation, or study-directed assignments occurred at any point. All bedside assessments and documentation were performed by the primary ICU nursing staff as part of routine clinical practice. Decisions regarding the type and progression of enteral nutrition—including the use of formula, human milk, or mixed regimens—were made solely by the congenital cardiac surgical and medical teams. The study did not alter any medical nutrition orders; rather, it evaluated the consistency with which nursing staff applied the standardized monitoring and reporting procedures.

### Implementation strategy and audit-and-feedback

The pathway was rolled out unit-wide using a standardized implementation approach. Prior to launch, bedside nurses received a structured education session covering the rationale for standardized feeding-tolerance surveillance, definitions of each monitoring indicator, and the predefined alert-trigger thresholds. Standardized documentation forms were distributed and integrated into routine nursing records. During the initial rollout period, charge nurses and nurse educators provided bedside support to reinforce consistent measurement techniques and documentation practices. Thereafter, the Nursing Department conducted periodic audits of documentation completeness and alert-trigger adherence, and aggregated feedback was provided to nursing staff during routine unit meetings to reinforce performance and address recurrent documentation issues.

### Protocol standardization

#### Standardization of risk assessment documentation

The standardized pathway incorporated a unified FI risk assessment form that required complete daily documentation. The form specified the essential EN monitoring elements, including abdominal girth, gastric residuals, emesis, and abdominal distension, and provided clear thresholds to guide interpretation. This structure ensured that all bedside nurses followed the same criteria when evaluating feeding tolerance.

#### Standardization of EN monitoring indicators

To improve consistency in surveillance, the protocol defined the frequency and method for each monitoring parameter. Abdominal girth measurements were recorded at least once to twice daily, and gastric residual volumes exceeding 50% of the previous feed were identified as a warning sign. Emesis and abdominal distension were documented using unified trigger criteria, reducing variability in symptom reporting across nursing staff.

#### Warning triggers and feeding-tolerance actions

The pathway outlined explicit conditions under which an event should be classified as FI and clarified the clinical thresholds for pausing vs. continuing EN. These criteria were designed to reduce ambiguity in bedside decision-making and to support timely recognition of intolerance. The protocol focused on improving the accuracy and uniformity of nursing assessments without superseding clinical judgment.

#### Recommendations for timing of EN initiation

The pathway encouraged early postoperative evaluation of EN readiness but did not impose mandatory initiation times. Decisions regarding the timing and method of EN initiation remained fully under the purview of the attending cardiac surgical and medical teams. Thus, the standardized protocol influenced nursing execution and monitoring practices while leaving medical feeding orders unchanged.

### Outcomes

#### Primary outcome

The primary outcome was the incidence of FI. FI was defined as the presence of one or more of the following criteria: (1) Gastric residual volume ≥50% of the previous feed; (2) Recurrent emesis; (3) Clinically significant abdominal distension; (4) An increase in abdominal girth of more than 2 cm within 24 h; (5) Interruption of enteral nutrition due to gastrointestinal intolerance. These components were selected to reflect clinically actionable signs routinely monitored in postoperative congenital heart disease care.

#### Secondary outcomes

Secondary outcomes captured broader aspects of feeding progression, gastrointestinal symptoms, and clinical recovery. These included the time to achieve full enteral feeding, defined as ≥100 mL/kg/day maintained for at least 24 h; the number of episodes of vomiting, abdominal distension, and gastric residuals exceeding threshold levels; and the total number of days during which enteral nutrition was held for gastrointestinal reasons. Additional clinical outcomes included the occurrence of necrotizing enterocolitis (Bell stage ≥ II) and bloody stools, duration of mechanical ventilation, length of ICU stay, and changes in weight-for-age z-scores. Nutritional status was further assessed by the proportion of infants meeting criteria for malnutrition (WAZ < −2).

### Data sources

Data were extracted from multiple components of the hospital's clinical documentation system to ensure comprehensive assessment of feeding-tolerance–related processes. Primary sources included routine nursing electronic records, dedicated enteral nutrition (EN) flow sheets, and standardized forms used for documenting abdominal girth and gastric residual volumes. Additional perioperative data—such as operative notes, postoperative progress records, anesthesia documentation, and cardiopulmonary bypass parameters—were obtained from the electronic medical record. These sources provided detailed information on both nursing execution and clinical status across the perioperative course.

### Data quality assurance

Quality control procedures were implemented to maintain the accuracy and completeness of extracted data. The Nursing Department conducted periodic audits of documentation quality, focusing on consistency and adherence to standard recording requirements. Records displaying missing key variables, conflicting entries, or incomplete EN monitoring information were excluded from analysis to avoid misclassification and ensure reliability of the study dataset.

### Statistical analysis

Continuous variables were summarized using means with standard deviations or medians with interquartile ranges, depending on distributional characteristics. Categorical variables were reported as frequencies and percentages. Between-group differences in the primary outcome (FI) were evaluated using *χ*^2^ tests or Fisher's exact tests when appropriate. Time to full enteral feeding was analyzed through Kaplan–Meier survival curves with comparisons made using the log-rank test. Counts of gastrointestinal symptoms, including vomiting, abdominal distension, and episodes of elevated gastric residuals, were modeled using negative binomial regression with ICU or hospital days included as the offset to account for variation in observation time.

To account for potential confounding without implying causal inference, multivariable logistic regression was used to assess factors associated with FI (yes/no), and Cox proportional hazards models were applied to evaluate time to full enteral feeding. Both models incorporated prespecified covariates based on clinical relevance: high surgical severity (STAT category 4–5), single-ventricle physiology, duration of cardiopulmonary bypass, peak postoperative lactate level, preoperative exposure to enteral nutrition, and postoperative ECMO use.

Several sensitivity analyses were performed to assess the robustness of the findings. These included repeating analyses after excluding infants with extreme ICU lengths of stay, restricting the cohort to those with complete follow-up through day of life 28, and conducting subgroup analyses stratified by early vs. delayed EN initiation (≤24 h vs. >24 h postoperatively). All statistical tests were two-sided, and a *P* value <0.05 was considered indicative of statistical significance. No correction for multiple comparisons was applied, and this limitation is acknowledged in the Discussion.

## Results

### Study population and cohort flow

**Figure 1:**
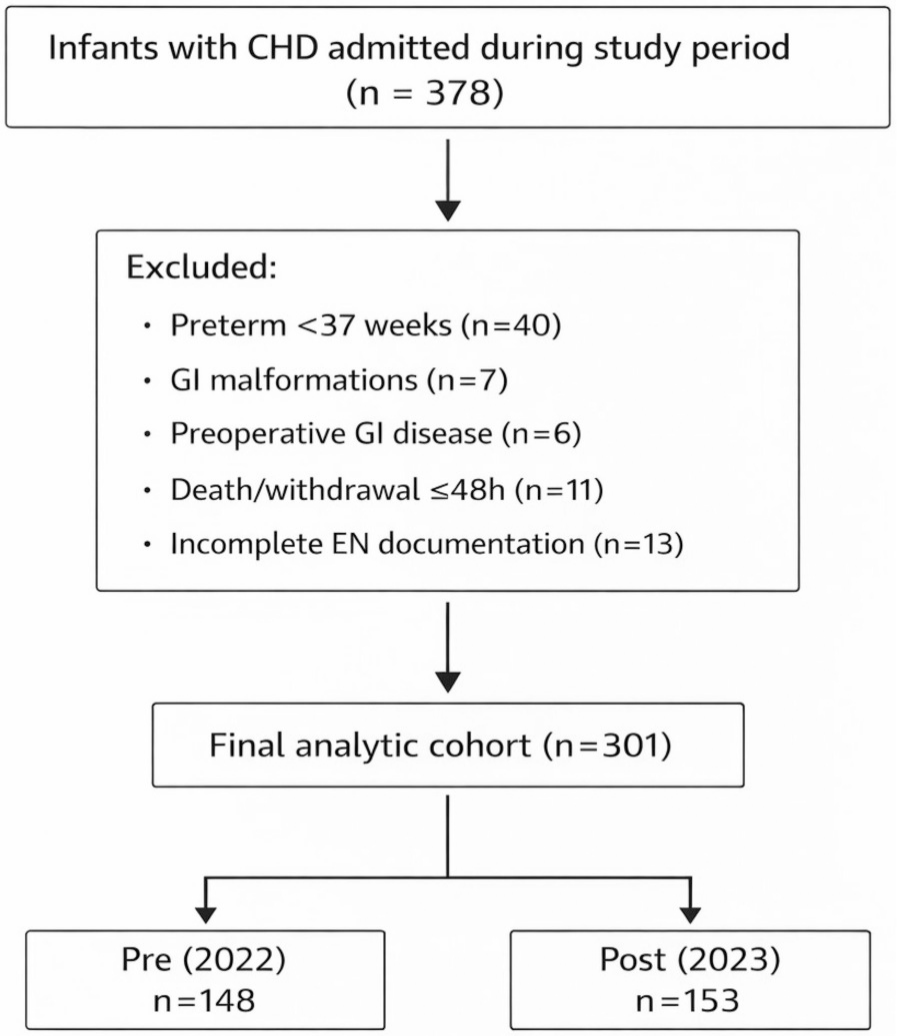
Flow diagram of cohort selection in the pre–post quality improvement study. Infants with congenital heart disease (CHD) admitted to the cardiac intensive care unit during the study period were screened for eligibility. Exclusion criteria included gestational age <37 weeks, major gastrointestinal malformations, preoperative necrotizing enterocolitis or severe gastrointestinal disease, death or withdrawal of care within 48 h postoperatively, and incomplete enteral nutrition documentation. A total of 301 infants met analytic inclusion criteria and were categorized into pre-implementation (2022, *n* = 148) and post-implementation (2023, *n* = 153) cohorts according to the timing of standardized feeding-tolerance pathway implementation.

Of 378 total infants with congenital heart disease admitted during the study period, 301 infants met analytic inclusion criteria (Figure 1). After excluding infants born <37 weeks (*n* = 40), those with major gastrointestinal malformations (*n* = 7), preoperative NEC or severe gastrointestinal disease (*n* = 6), postoperative mortality or withdrawal within 48 h (*n* = 11), and incomplete enteral feeding documentation (*n* = 13), 301 infants met analytic inclusion criteria. A total of 301 postoperative CHD infants were included, comprising 148 in the pre-implementation cohort (2022) and 153 in the post-implementation cohort (2023) (Supplementary Figure 1).

### Baseline characteristics of infants in the pre- and post-implementation cohorts

Demographic and nutritional parameters were well balanced between cohorts, with no significant differences observed (all *P* > 0.05) (Table 1). Severity of cardiac pathology did not differ. The proportion of single-ventricle physiology was nearly identical (24.32% vs. 23.53%), and STAT 4–5 categories accounted for 22.30% and 21.57% in the two cohorts (*P* = 0.719), suggesting comparable anatomical and surgical risk.

**Table 1:** Baseline demographic, cardiac, and perioperative characteristics of infants in the pre- and post-implementation cohorts.

Characteristic	Pre-implementation (*n* = 148)	Post-implementation (*n* = 153)	*P* value
Nasogastric tube, *n* (%)	148 (100)	153 (100)	—
Sex, male, *n* (%)	84 (56.76)	89 (58.17)	0.812
Age at surgery, days, median (IQR)	72.50 (41.00–118.00)	75.00 (43.00–120.00)	0.594
Body weight at surgery, kg, mean ± SD	4.12 ± 0.82	4.15 ± 0.85	0.764
Birth weight, kg, mean ± SD	3.05 ± 0.47	3.07 ± 0.51	0.708
WAZ at admission, mean ± SD	−1.12 ± 0.64	−1.08 ± 0.59	0.641
Cardiac lesion type			0.853
Biventricular repair, *n* (%)	112 (75.68)	117 (76.47)	—
Single-ventricle physiology, *n* (%)	36 (24.32)	36 (23.53)	—
STAT surgical category, *n* (%)			0.719
STAT 1–2	52 (35.14)	57 (37.25)	—
STAT 3	63 (42.57)	63 (41.18)	—
STAT 4–5	33 (22.30)	33 (21.57)	—
Cardiopulmonary bypass duration, min, median (IQR)	92.00 (74.00–124.00)	94.00 (75.00–126.00)	0.683
Aortic cross-clamp time, min, median (IQR)	52.00 (41.00–70.00)	53.00 (41.00–72.00)	0.776
Peak postoperative lactate, mmol/L, mean ± SD	4.18 ± 1.26	4.11 ± 1.22	0.672
Vasoactive–inotropic score, median (IQR)	12.00 (8.00–17.00)	12.00 (8.00–18.00)	0.859
Nitric oxide therapy, *n* (%)	14 (9.46)	17 (11.11)	0.651
ECMO use, *n* (%)	4 (2.70)	5 (3.27)	0.787
Mechanical ventilation duration, days, median (IQR)	3.00 (2.00–5.00)	3.00 (2.00–5.00)	0.942
Preoperative enteral feeding, *n* (%)	121 (81.76)	126 (82.35)	0.903
Preoperative feeding type			0.717
Human milk	102 (68.92)	104 (67.97)	—
Formula	46 (31.08)	49 (32.03)	—

Values are presented as mean ± SD, median (IQR), or *n* (%), as appropriate.

All between-group comparisons yielded non-significant differences (*P* > 0.05), indicating balanced demographic, cardiac, and perioperative profiles at baseline.

ECMO, extracorporeal membrane oxygenation; WAZ, weight-for-age Z score; STAT, Society of Thoracic Surgeons–European Association for Cardio-Thoracic Surgery risk category.

Perioperative stress markers displayed similar trends. Cardiopulmonary bypass duration [92.00 (74.00–124.00) vs. 94.00 (75.00–126.00) min, *P* = 0.683] and aortic cross-clamp time [52.00 (41.00–70.00) vs. 53.00 (41.00–72.00) min, *P* = 0.776] were nearly overlapping. Peak postoperative lactate remained aligned (4.18 ± 1.26 vs. 4.11 ± 1.22 mmol/L, *P* = 0.672), as did vasoactive requirements [median VIS 12.00 (8.00–17.00) vs. 12.00 (8.00–18.00), *P* = 0.859]. Nitric oxide use (9.46% vs. 11.11%, *P* = 0.651) and ECMO initiation (2.70% vs. 3.27%, *P* = 0.787) were infrequent and proportionally equivalent.

Early postoperative respiratory support trajectories were equally balanced. Median mechanical ventilation duration was 3.00 days in both groups (IQR 2.00–5.00, *P* = 0.942). Preoperative enteral feeding exposure (81.76% vs. 82.35%, *P* = 0.903) and feeding type—human milk (68.92% vs. 67.97%) vs. formula—showed no meaningful divergence.

### Implementation of the unit-wide feeding tolerance protocol

Completion of the feeding intolerance risk assessment increased from 48.65% to 90.85%, and overall enteral nutrition documentation completeness rose from 68.42% ± 11.07% to 91.73% ± 9.84% (both *P* < 0.001) (Table 2). Adherence to predefined alert criteria, including recording of gastric residuals, emesis, and abdominal distension, increased from 54.73% to 92.16% (*P* < 0.001). Routine monitoring frequency demonstrated a parallel shift, with abdominal girth assessment increasing from 1.32 ± 0.41 to 1.88 ± 0.46 times per day and gastric residual evaluations from 1.15 ± 0.38 to 1.72 ± 0.44 times per day (both *P* < 0.001). Median time to enteral nutrition initiation was earlier in the post-implementation cohort [27.00 h (IQR 20.00–38.00) vs. 34.00 h (26.00–45.00), *P* = 0.014], and the proportion receiving enteral feeding within 48 h increased from 55.41% to 79.08%.

**Table 2:** Implementation fidelity and feeding initiation indicators before and after protocol standardization.

Indicator	Pre-implementation (*n* = 148)	Post-implementation (*n* = 153)	*P* value
Completion of FI nursing risk assessment forms, *n* (%)	72 (48.65)	139 (90.85)	<0.001
Completeness of EN nursing documentation^a^, %	68.42 ± 11.07	91.73 ± 9.84	<0.001
Documentation of predefined EN alert criteria^b^, *n* (%)	81 (54.73)	141 (92.16)	<0.001
Frequency of abdominal girth measurement, per day, mean ± SD	1.32 ± 0.41	1.88 ± 0.46	<0.001
Frequency of gastric residual evaluation, per day, mean ± SD	1.15 ± 0.38	1.72 ± 0.44	<0.001
Time to initiation of enteral feeding, hours, median (IQR)	34.00 (26.00–45.00)	27.00 (20.00–38.00)	0.014
Initiation of EN within 48 h postoperatively, *n* (%)	82 (55.41)	121 (79.08)	<0.001

^a^ EN documentation completeness included abdominal girth, gastric residuals, stool passage, vomiting episodes, and abdominal distension entries recorded per protocol.

^b^ EN alert criteria comprised predefined triggers: gastric residual ≥50% of previous feed, emesis, clinically significant abdominal distension, or new-onset feeding intolerance signs.

### Incidence of feeding intolerance

The incidence of feeding intolerance decreased from 42.57% in the pre-implementation cohort to 24.18% in the post-implementation cohort (RR 0.57, 95% CI 0.41–0.79; *P* = 0.001). EN cessation due to intolerance was similarly reduced (24.32% vs. 9.80%; RR 0.40, 95% CI 0.22–0.72; *P* = 0.003) (Table 3).

**Table 3:** Incidence of feeding intolerance in the pre- and post-implementation cohorts.

Outcome	Pre-implementation (*n* = 148)	Post-implementation (*n* = 153)	Effect estimate	*P* value
Feeding intolerance, *n* (%)	63 (42.57)	37 (24.18)	RR 0.57 (95% CI 0.41–0.79)	0.001
Crude OR (95% CI)	—	—	OR 0.43 (95% CI 0.26–0.70)	0.001
Clinical FI resolution without EN cessation, *n* (%)	27 (18.24)	41 (26.80)	RR 1.47 (95% CI 1.01–2.14)	0.046
EN cessation due to FI, *n* (%)	36 (24.32)	15 (9.80)	RR 0.40 (95% CI 0.22–0.72)	0.003

RR, risk ratio; OR, odds ratio; CI, confidence interval.

Feeding intolerance (FI) was defined as meeting ≥1 of the following: gastric residual ≥50% of previous feed, recurrent emesis, clinically significant abdominal distension, abdominal girth escalation >2 cm over 24 h, or interruption of enteral nutrition due to gastrointestinal intolerance. Crude comparisons were performed using *χ*^2^ tests. All estimates reflect unadjusted risk differences between time-defined care pathways rather than assigned interventions.

The multivariable model adjusted for prespecified clinical severity indicators, including STAT category, single-ventricle physiology, CPB duration, peak lactate, preoperative enteral nutrition exposure, and ECMO use, to address confounding by postoperative illness burden and hemodynamic compromise. In multivariable analysis, assignment to the post-implementation period remained independently associated with lower odds of feeding intolerance (aOR 0.48, 95% CI 0.29–0.79; *P* = 0.004) (Table 4).

**Table 4:** Multivariable logistic regression analysis of factors associated with feeding intolerance.

Variable	Adjusted OR (aOR)	95% CI	*P* value
Post-implementation cohort^a^	0.48	0.29–0.79	0.004
STAT category (4–5 vs. 1–3)	1.82	1.07–3.15	0.028
Single-ventricle physiology	1.56	0.93–2.72	0.094
Cardiopulmonary bypass time, per 30-min increase	1.19	0.98–1.42	0.078
Peak postoperative lactate, per 1 mmol/L increase	1.21	1.02–1.46	0.031
Preoperative enteral feeding (yes vs. no)	0.79	0.46–1.36	0.392
ECMO use (yes vs. no)	2.03	0.87–5.41	0.111

^a^ Reference group: pre-implementation cohort.

Variables included in the multivariable model were selected *a priori* based on clinical relevance: STAT surgical category, single-ventricle physiology, cardiopulmonary bypass time, peak lactate, preoperative enteral nutrition exposure, and ECMO use. Feeding intolerance (FI) was defined as meeting ≥1 predefined gastrointestinal intolerance criteria resulting in feeding interruption, escalation, or symptomatic feeding delay. VIF diagnostics showed no collinearity concerns (all <2.0), and model fit was acceptable (Hosmer–Lemeshow *P* = 0.62).

### Secondary outcomes related to enteral feeding progression and gastrointestinal symptoms

The time to full enteral feeding was shorter in the post-implementation cohort [6.00 days (IQR 5.00–9.00) vs. 9.00 days (7.00–12.00); HR 1.48, 95% CI 1.11–1.97; *P* = 0.008]. Gastrointestinal symptom burden also declined, including vomiting episodes (IRR 0.62, 95% CI 0.44–0.88; *P* = 0.009) and abdominal distension (IRR 0.59, 95% CI 0.38–0.90; *P* = 0.015). Feeds held for GI-related concerns were reduced from a median of 2.00 to 1.00 days (IRR 0.55, 95% CI 0.36–0.83; *P* = 0.006) (Table 5).

**Table 5:** Secondary enteral feeding progression and gastrointestinal symptom outcomes in pre- and post-implementation cohorts.

Outcome	Pre-implementation (*n* = 148)	Post-implementation (*n* = 153)	Effect estimate	*P* value
Time to full enteral feeding, days, median (IQR)	9.00 (7.00–12.00)	6.00 (5.00–9.00)	HR 1.48 (95% CI 1.11–1.97)^a^	0.008
Vomiting episodes, per patient, median (IQR)	2.00 (1.00–4.00)	1.00 (0.00–3.00)	IRR 0.62 (95% CI 0.44–0.88)	0.009
Abdominal distension episodes, per patient, median (IQR)	1.00 (0.00–2.00)	0.00 (0.00–1.00)	IRR 0.59 (95% CI 0.38–0.90)	0.015
Feeds held due to GI intolerance, days, median (IQR)	2.00 (1.00–4.00)	1.00 (0.00–2.00)	IRR 0.55 (95% CI 0.36–0.83)	0.006
Elevated gastric residuals (>50% prior feed), *n* (%)	58 (39.19)	34 (22.22)	RR 0.57 (95% CI 0.39–0.82)	0.004
NEC (Bell stage ≥ II), *n* (%)	4 (2.70)	2 (1.31)	—	0.423
Bloody stools requiring NEC rule-out, *n* (%)	11 (7.43)	6 (3.92)	—	0.168

HR, hazard ratio; IRR, incidence rate ratio; RR, risk ratio; CI, confidence interval.

Full enteral feeding was defined as ≥100 mL/kg/day sustained for ≥24 h without feeding interruption. IRR estimates were derived from negative binomial regression models adjusted for CICU length of stay and STAT category. NEC events were rare; therefore, descriptive reporting was prioritized over inferential testing.

^a^ Higher HR reflects earlier attainment of full enteral feeding in the post-implementation cohort.

Kaplan–Meier analysis demonstrated a significantly shorter time to full enteral feeding in the post-implementation cohort compared with the pre-implementation cohort (Supplementary Figure 2). The probability of remaining below full enteral feeding declined more rapidly following protocol implementation, consistent with an earlier attainment of nutritional goals (log-rank *P* = 0.008).

### Clinical recovery outcomes and nutritional status

ICU length of stay was modestly shorter in the post-implementation period (11.00 vs. 13.00 days; HR 1.31, 95% CI 1.02–1.62; *P* = 0.031). Nutritional decline over admission was attenuated following implementation, reflected by improved ΔWAZ (−0.04 vs. −0.22; *P* = 0.004) and a lower malnutrition rate at discharge (18.95% vs. 31.08%; OR 0.51, 95% CI 0.31–0.86; *P* = 0.011) (Table 6 and Supplementary Figure 2).

**Table 6:** Clinical recovery and nutritional outcomes in pre- and post-implementation cohorts.

Outcome	Pre-implementation (*n* = 148)	Post-implementation (*n* = 153)	Effect estimate	*P* value
Invasive ventilation, days, median (IQR)	5.00 (3.00–8.00)	4.00 (2.00–6.00)	HR 1.27 (95% CI 0.98–1.61)	0.072
ICU length of stay, days, median (IQR)	13.00 (10.00–18.00)	11.00 (8.00–15.00)	HR 1.31 (95% CI 1.02–1.62)	0.031
Hospital length of stay, days, median (IQR)	23.00 (18.00–30.00)	20.00 (16.00–27.00)	HR 1.18 (95% CI 0.94–1.46)	0.142
WAZ at admission, mean ± SD	−1.12 ± 0.64	−1.08 ± 0.59	—	0.641
WAZ at DOL 28/discharge, mean ± SD	−1.34 ± 0.71	−1.12 ± 0.63	—	0.022
ΔWAZ (discharge − admission), mean ± SD	−0.22 ± 0.38	−0.04 ± 0.32	—	0.004
Malnutrition (WAZ < −2), *n* (%)	46 (31.08)	29 (18.95)	OR 0.51 (95% CI 0.31–0.86)	0.011
Re-intubation rate, *n* (%)	11 (7.43)	7 (4.58)	—	0.281
No major feeding-related complications, *n* (%)	131 (88.51)	142 (92.81)	—	0.184
Postoperative mortality (>48 h), *n* (%)	**3** (**2.03)**	**2** (**1.31)**	—	0.682

ΔWAZ indicates change in weight-for-age z-score between admission and DOL 28 (or discharge if earlier). HR for hospital/ICU stay reflects time-to-discharge/time-to-extubation directionality rather than causal therapeutic interpretation. Malnutrition defined as WAZ < −2 per WHO growth reference. Feeding-related complications include NEC (Bell ≥ II), persistent bloody stools requiring clinical intervention, and EN cessation >72 h.

### Sensitivity and subgroup analyses

Subgroup findings were directionally consistent with the primary analysis. Among infants with high surgical complexity (STAT 4–5 or single-ventricle physiology), the post-implementation cohort exhibited lower feeding intolerance (aOR 0.54, 95% CI 0.31–0.96) and shorter time to full enteral feeding (HR 1.42, 95% CI 1.03–1.97), although estimate precision was reduced given the smaller denominator (Supplementary Table S1). For infants fed within 24 h postoperatively, feeding intolerance remained less frequent in the post-implementation cohort (aOR 0.47, 95% CI 0.24–0.94), whereas effect attenuation was observed in those initiated after 24 h (aOR 0.61, 95% CI 0.35–1.08). Time-to-full enteral feeding also favored implementation in early-fed infants (HR 1.55, 95% CI 1.10–2.23) with a modestly smaller magnitude in delayed-feeding strata (Supplementary Table S2). Across all sensitivity frameworks, including exclusion of prolonged CICU stays, restriction to complete DOL 28 Follow-up, and adjustment for vasoactive burden, cohort-level differences remained stable (aOR 0.46–0.52) (Supplementary Table S3).

## Discussion

The implementation of a standardized feeding-tolerance pathway was associated with several favorable trends across perioperative enteral nutrition outcomes. The incidence of feeding intolerance declined in the post-implementation cohort, aligning with improved consistency in monitoring practices. Infants achieved full enteral feeding earlier—a shift from a median of nine to six days—reflecting greater procedural reliability and more timely detection of tolerance. Gastrointestinal intolerance symptoms, including vomiting, elevated gastric residuals, and abdominal distension, occurred less frequently and paralleled the increase in structured documentation and warning-trigger adherence. Nutritional trajectories also demonstrated a more stable profile, with smaller declines in weight-for-age z-scores and a lower prevalence of malnutrition. Together, these trends indicate that improving the consistency of feeding-tolerance monitoring may hold practical value for supporting safer nutritional progression and stabilizing postoperative recovery in infants with congenital heart disease.

Infants with CHD represent a population with intrinsically high vulnerability to gastrointestinal dysfunction (20, 21). In many forms of ductal-dependent or parallel circulatory physiology, rapid closure of the ductus arteriosus can precipitate hemodynamic deterioration, leading to intestinal hypoperfusion and impaired oxygen delivery (22–24). Severe cardiac malformations further compound this risk by contributing to heart failure, altered gut motility, increased metabolic demands, and reduced nutritional intake, all of which place CHD infants at heightened risk for preoperative malnutrition (25–27). Feeding intolerance often emerges as a manifestation of these physiological disturbances, driven by prolonged ischemia and hypoxia of the gastrointestinal tract (16, 28, 29). If unrecognized, FI can compromise nutrient absorption, delay recovery, and predispose infants to complications, including necrotizing enterocolitis (30, 31). Although FI is typically assessed using signs such as gastric residuals, abdominal distension, and emesis, symptom definitions vary, and additional indicators—such as circulatory instability or elevated serum lactate—may reflect impaired splanchnic perfusion (32, 33). However, the absence of well-defined lactate thresholds limits its diagnostic utility in routine care. Against this physiological background, consistent and accurate monitoring becomes essential for timely detection of early intolerance, underscoring the clinical relevance of process-based improvements evaluated in the present study.

A central observation in this study is the role of nursing execution consistency in the identification of feeding intolerance. More uniform assessment and documentation practices appeared to reduce delays in recognizing early signs of intolerance and may have limited unnecessary interruptions of enteral nutrition. The completeness of key monitoring records—including abdominal girth, gastric residuals, and emesis—improved from approximately 68%–92%, narrowing the margin for missed or ambiguous events.

The applicability of standardized EN monitoring to higher-risk subgroups, such as infants with STAT category 4–5 procedures or single-ventricle physiology, was also reflected in the findings. Although these infants continued to demonstrate an elevated susceptibility to feeding intolerance, the magnitude of variability appeared more controlled in the post-implementation period. Given the well-recognized vulnerability of single-ventricle patients to perfusion instability, a consistent monitoring pathway may help avoid unnecessary interruptions of enteral nutrition triggered by ambiguous or inconsistently interpreted signs. The overall pattern aligns with existing literature in pediatric cardiac critical care, which highlights that uniformity in gastrointestinal monitoring practices contributes more reliably to safe nutrition advancement than attempts to accelerate feeding initiation (12, 34–38).

The modest and directionally consistent reduction in time to full EN likely reflects fewer delays attributable to inconsistent monitoring or unverified concerns. In this context, the clinical relevance lies not in promoting earlier feeding, but in minimizing unplanned interruptions that stem from documentation gaps or variable recognition of gastrointestinal signs. Such improvements in process reliability may help maintain continuity of nutritional advancement. The smaller decline in weight-for-age z-scores observed in the post-implementation cohort (from −0.22 to −0.04) suggests a pattern of reduced in-hospital nutritional loss. This trend is consistent with fewer disruptions in enteral feeding and greater continuity of caloric delivery, both of which may limit the degree of catabolism typically seen during postoperative recovery. The post-implementation cohort exhibited directionally shorter durations of mechanical ventilation and ICU stay. More reliable monitoring and fewer unplanned interruptions of enteral nutrition may contribute to a smoother postoperative course.

The robustness of these findings was supported through subgroup and sensitivity analyses. Across higher-risk subgroups—including infants with STAT category 4–5 procedures and single-ventricle physiology—the direction of associations remained consistent, with no reversal in trends related to feeding intolerance or feeding progression. Sensitivity analyses that excluded infants with extreme ICU lengths of stay yielded similar results, further reinforcing the stability of the observed patterns. These aligned trends do not imply a causal effect but rather indicate that improved process consistency and monitoring reliability may have jointly contributed to the more favorable trajectory of FI-related outcomes.

Although the present study focuses on infants undergoing congenital heart surgery, the observed benefits of standardized feeding-tolerance monitoring may have broader relevance across age groups. Similar challenges related to variability in enteral access management and feeding progression have been reported in adult cardiac populations. A recent retrospective cohort study by Rouhi et al. demonstrated that enteral access outcomes in adults hospitalized with cardiac disease were influenced not only by patient characteristics but also by care processes and monitoring practices (39). These findings align with the present results, suggesting that structured assessment frameworks and consistent documentation may improve the reliability of enteral nutrition delivery irrespective of patient age. While physiological differences between pediatric and adult populations must be acknowledged, the shared importance of process standardization highlights a potential cross-disciplinary application of implementation-focused nutritional care strategies.

The findings offer several implications for nursing practice, particularly within postoperative congenital heart care. Standardizing the processes used to identify feeding intolerance may help reduce diagnostic overreactions, such as unnecessary interruptions of enteral nutrition prompted by inconsistent documentation or subjective interpretation. In high-risk surgical populations, the completeness and reliability of nursing records constitute essential foundational information for day-to-day decisions about feeding progression.

Several limitations should be considered when interpreting these findings. The single-center design may limit generalizability, as local workflow culture and staffing patterns could influence the applicability of the standardized pathway in other settings. The study did not involve randomization or controlled assignment of interventions, and therefore the results should not be interpreted as causal. Because the FI definition incorporated multiple nurse-documented indicators, measurement accuracy was inherently dependent on documentation quality, which may introduce misclassification despite audit procedures. Longer-term nutritional outcomes were not assessed, preventing evaluation of whether the observed trends in in-hospital nutritional stability persist beyond discharge. In addition, differences in enteral feeding formulas were not analyzed, as medical feeding prescriptions were independently determined and lay outside the scope of the nursing pathway. Future work should explore whether the relationship between pathway consistency and FI recognition holds across diverse institutions, particularly those with different staffing structures or documentation systems. Multicenter quality improvement collaborations may help clarify how local workflow factors influence nursing execution and monitoring reliability.

## Conclusion

In this quality improvement analysis, the implementation of a standardized feeding-tolerance pathway was associated with a lower incidence of feeding intolerance and a trend toward earlier attainment of full enteral nutrition. Improvements in documentation completeness and monitoring consistency paralleled these clinical patterns, underscoring the importance of reliable nursing execution in postoperative nutritional management for infants with congenital heart disease. Taken together, the findings support the applicability of process-focused quality improvement strategies in this population. Nevertheless, the observed trends require validation in multicenter settings, with longer follow-up and integration of electronic EN monitoring systems, to minimize the influence of site-specific documentation practices and ensure broader generalizability.

## Data Availability

The original contributions presented in the study are included in the article/Supplementary Material, further inquiries can be directed to the corresponding author/s.
