# Supplementary material for: Impact of a unit-wide feeding tolerance management protocol on enteral feeding outcomes in infants with congenital heart disease: a pre–post quality improvement cohort study
Source: Front Rehabil Sci. 2026 Mar 27;7:1765642. doi: 10.3389/fresc.2026.1765642 (PMC13066188; doi:10.3389/fresc.2026.1765642)
Supplement: Supplementary file 1 [file Datasheet1.pdf]

```
"""
CHD EN Feeding Intolerance Study
Python Analysis Script
--------------------------------
This script reproduces the main analyses reported in the manuscript:
- Descriptive statistics and baseline comparison
- Incidence of feeding intolerance (FI)
- Group comparisons for FI and secondary outcomes
- Multivariable logistic regression for FI
- (Optional) Time-to-event analysis (time to full EN) with Cox regression
- Negative binomial models for gastrointestinal symptom counts
- Sensitivity and subgroup analyses

Author: (fill in your name)
Contact: (fill in your email)

Software requirements:
- Python >= 3.8
- pandas
- numpy
- scipy
- statsmodels
(Optional)
- lifelines  (for KM/Cox, if you want to draw curves)
"""

import pandas as pd
import numpy as np

from scipy import stats
import statsmodels.api as sm
import statsmodels.formula.api as smf
import statsmodels.discrete.discrete_model as discrete

################################################################################
# 0. LOAD DATA
################################################################################

# Adjust path if needed
DATA_PATH = "CHD_EN_rawdata_301.xlsx"

df = pd.read_excel(DATA_PATH)

print("Data shape:", df.shape)
print("Columns:", df.columns.tolist())

# Recode cohort as label: 0=Pre, 1=Post
df["Cohort_label"] = df["Cohort"].map(
    {0: "Pre-implementation", 1: "Post-implementation"}
)

################################################################################
# 1. DESCRIPTIVE STATISTICS & BASELINE COMPARISON (Table 1)
################################################################################

def summarize_continuous(var: str) -> pd.DataFrame:
    """Summary by cohort for continuous variables."""
    g = df.groupby("Cohort")[var]
    summary = g.agg(["mean", "std", "median", "min", "max"])
    return summary

def chisq_by_cohort(var: str):
    """Chi-square test for categorical variable by cohort."""
    ct = pd.crosstab(df[var], df["Cohort"])
    chi2, p, dof, exp = stats.chi2_contingency(ct)
    return ct, chi2, p

baseline_continuous = [
    "Age_surgery_days",
    "Birth_weight_kg",
    "Surgery_weight_kg",
    "WAZ_admission",
    "CPB_time_min",
    "Lactate_peak",
]
```

```
baseline_categorical = [
    "Sex",
    "STAT_high",
    "Single_ventricle",
    "NO_use",
    "ECMO",
    "Preop_EN",
]

print("\n=== Baseline continuous variables by cohort ===")
for var in baseline_continuous:
    print(f"\nVariable: {var}")
    print(summarize_continuous(var))

print("\n=== Baseline categorical variables by cohort ===")
for var in baseline_categorical:
    ct, chi2, p = chisq_by_cohort(var)
    print(f"\nVariable: {var}")
    print(ct)
    print(f"Chi-square p-value: {p:.3f}")

###############################################################################
# 2. PRIMARY OUTCOME: FEEDING INTOLERANCE (FI)
###############################################################################

print("\n=== Primary outcome: FI incidence by cohort ===")

ct_fi = pd.crosstab(df["FI"], df["Cohort"])
print(ct_fi)

# Proportions
fi_pre = ct_fi.loc[1, 0] / ct_fi[0].sum()
fi_post = ct_fi.loc[1, 1] / ct_fi[1].sum()
print(f"FI incidence - Pre: {fi_pre:.3f}, Post: {fi_post:.3f}")

# Risk ratio (RR) for Post vs Pre
rr = fi_post / fi_pre
# Approximate CI for log(RR)
se_log_rr = np.sqrt(
    1 / ct_fi.loc[1, 0] - 1 / ct_fi[0].sum()
    + 1 / ct_fi.loc[1, 1] - 1 / ct_fi[1].sum()
)
ci_low = np.exp(np.log(rr) - 1.96 * se_log_rr)
ci_high = np.exp(np.log(rr) + 1.96 * se_log_rr)
print(f"RR (Post vs Pre): {rr:.2f} (95% CI {ci_low:.2f}-{ci_high:.2f})")

# Chi-square test
chi2, p, dof, exp = stats.chi2_contingency(ct_fi)
print(f"Chi-square p-value for FI by cohort: {p:.3f}")

###############################################################################
# 3. MULTIVARIABLE LOGISTIC REGRESSION FOR FI (Table 4)
###############################################################################

print("\n=== Multivariable logistic regression for FI ===")

covariates = [
    "Cohort",           # 0=Pre, 1=Post
    "STAT_high",
    "Single_ventricle",
    "CPB_time_min",
    "Lactate_peak",
    "Preop_EN",
    "ECMO",
]

formula_fi = (
    "FI ~ Cohort + STAT_high + Single_ventricle + "
    "CPB_time_min + Lactate_peak + Preop_EN + ECMO"
)

model_logit = smf.logit(formula=formula_fi, data=df).fit(disp=False)
print(model_logit.summary())
```

```python
# Adjusted ORs and 95% CI
params = model_logit.params
conf = model_logit.conf_int()
or_table = pd.DataFrame(
    {
        "OR": np.exp(params),
        "CI_low": np.exp(conf[0]),
        "CI_high": np.exp(conf[1]),
        "p_value": model_logit.pvalues,
    }
)
print("\nAdjusted ORs (logistic regression):")
print(or_table)

###############################################################################
# 4. OPTIONAL: KM & COX ANALYSIS FOR TIME TO FULL EN
###############################################################################
# If you have lifelines installed locally, you can uncomment this block:

"""
from lifelines import KaplanMeierFitter, CoxPHFitter

print("\\n=== Time to full EN: KM and Cox models ===")

T = df["Full_EN_day"]
E = np.ones(len(df), dtype=int)

kmf = KaplanMeierFitter()

for cohort_val, label in [(0, "Pre-implementation"), (1, "Post-implementation")]:
    mask = df["Cohort"] == cohort_val
    kmf.fit(T[mask], event_observed=E[mask], label=label)
    kmf.plot_survival_function()

cox_df = df[["Full_EN_day"] + covariates].copy()
cox_df = cox_df.rename(columns={"Full_EN_day": "T"})
cph = CoxPHFitter()
cph.fit(cox_df, duration_col="T", event_col=None)
print(cph.summary)
"""

###############################################################################
# 5. SECONDARY OUTCOMES: GI SYMPTOMS & EN INTERRUPTION (Table 5)
###############################################################################

print("\n=== Secondary outcomes by cohort ===")

sec_vars = [
    "Full_EN_day",
    "Vomiting_count",
    "Distension_count",
    "EN_stop_days",
    "Elevated_GRV",
]

for var in sec_vars:
    print(f"\nVariable: {var}")
    print(df.groupby("Cohort")[var].describe())

print("\n=== Negative binomial models for GI symptoms ===")

# Offset for ICU LOS
df["log_ICU_LOS"] = np.log(df["ICU_LOS_days"])

for outcome in ["Vomiting_count", "Distension_count"]:
    print(f"\nOutcome: {outcome}")
    X = sm.add_constant(
        pd.DataFrame(
            {
                "Cohort": df["Cohort"],
                "log_ICU_LOS": df["log_ICU_LOS"],
            }
        )
    )
    y = df[outcome]
```

```
    model_nb = discrete.NegativeBinomial(endog=y, exog=X)
    res_nb = model_nb.fit(disp=False)
    print(res_nb.summary())

###############################################################################
# 6. CLINICAL RECOVERY & NUTRITIONAL OUTCOMES (Table 6)
###############################################################################

print("\n=== Clinical recovery and nutritional outcomes by cohort ===")

recovery_vars = [
    "MV_days",
    "ICU_LOS_days",
    "Delta_WAZ",
]

for var in recovery_vars:
    print(f"\nVariable: {var}")
    print(df.groupby("Cohort")[var].describe())

print("\n=== Linear model for Delta_WAZ (exploratory) ===")

formula_dwaz = (
    "Delta_WAZ ~ Cohort + STAT_high + Single_ventricle + "
    "CPB_time_min + Lactate_peak"
)
model_dwaz = smf.ols(formula=formula_dwaz, data=df).fit()
print(model_dwaz.summary())

###############################################################################
# 7. SENSITIVITY & SUBGROUP ANALYSES
###############################################################################

print("\n=== Sensitivity analysis: excluding extreme ICU LOS ===")
q99 = df["ICU_LOS_days"].quantile(0.99)
df_sens = df[df["ICU_LOS_days"] <= q99].copy()
print("Shape after excluding extreme ICU LOS:", df_sens.shape)

model_logit_sens = smf.logit(formula=formula_fi, data=df_sens).fit(disp=False)
print("\nLogistic model for FI in sensitivity cohort:")
print(model_logit_sens.summary())

print("\n=== Subgroup analysis: high-risk STAT (4–5) ===")
df_high = df[df["STAT_high"] == 1].copy()
print("High-risk STAT sample size:", df_high.shape[0])
ct_fi_high = pd.crosstab(df_high["FI"], df_high["Cohort"])
print(ct_fi_high)

print("\n=== Subgroup analysis: EN start time ≤24 h vs >24 h ===")
df["EN_early"] = (df["EN_start_hours"] <= 24).astype(int)
ct_early = pd.crosstab(df["FI"], df["EN_early"])
print(ct_early)

###############################################################################
# 8. EXPORT KEY TABLES (OPTIONAL)
###############################################################################

with pd.ExcelWriter("CHD_EN_analysis_outputs.xlsx") as writer:
    ct_fi.to_excel(writer, sheet_name="FI_by_cohort")
    or_table.to_excel(writer, sheet_name="Logistic_ORs")
    df.groupby("Cohort")[sec_vars].describe().to_excel(
        writer, sheet_name="Secondary_outcomes"
    )
    df.groupby("Cohort")[recovery_vars].describe().to_excel(
        writer, sheet_name="Recovery_outcomes"
    )

print("\nAnalysis completed. Key model outputs saved to 'CHD_EN_analysis_outputs.xlsx'.")
```
