# Supplementary material for: Impact of a unit-wide feeding tolerance management protocol on enteral feeding outcomes in infants with congenital heart disease: a pre–post quality improvement cohort study
Source: Front Rehabil Sci. 2026 Mar 27;7:1765642. doi: 10.3389/fresc.2026.1765642 (PMC13066188; doi:10.3389/fresc.2026.1765642)
Supplement: Supplementary file 2 [file Table1.docx]

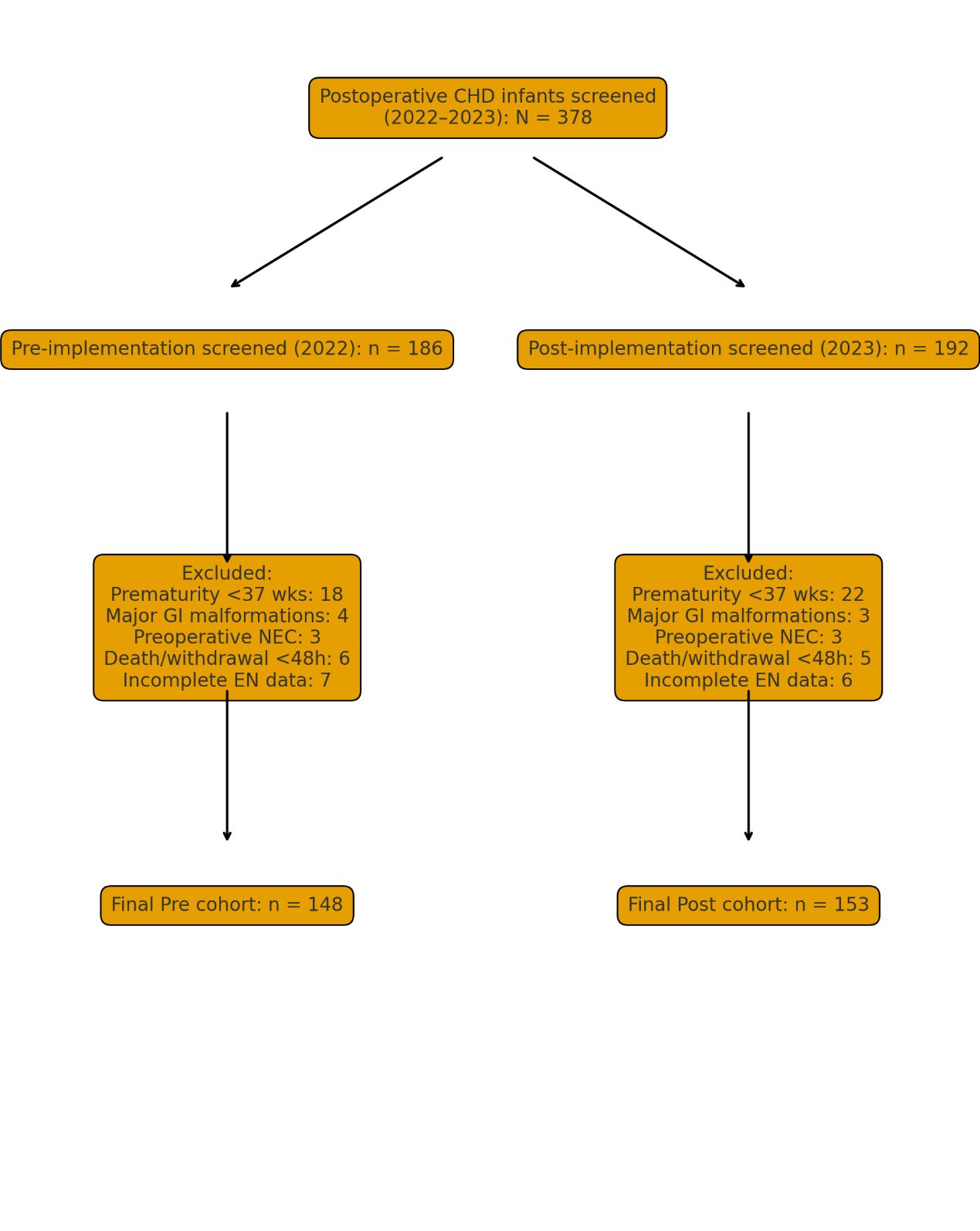


Supplementary Figure 1. Flow of cohort inclusion before and after unit-wide feeding tolerance protocol standardization. No participant-level allocation occurred.


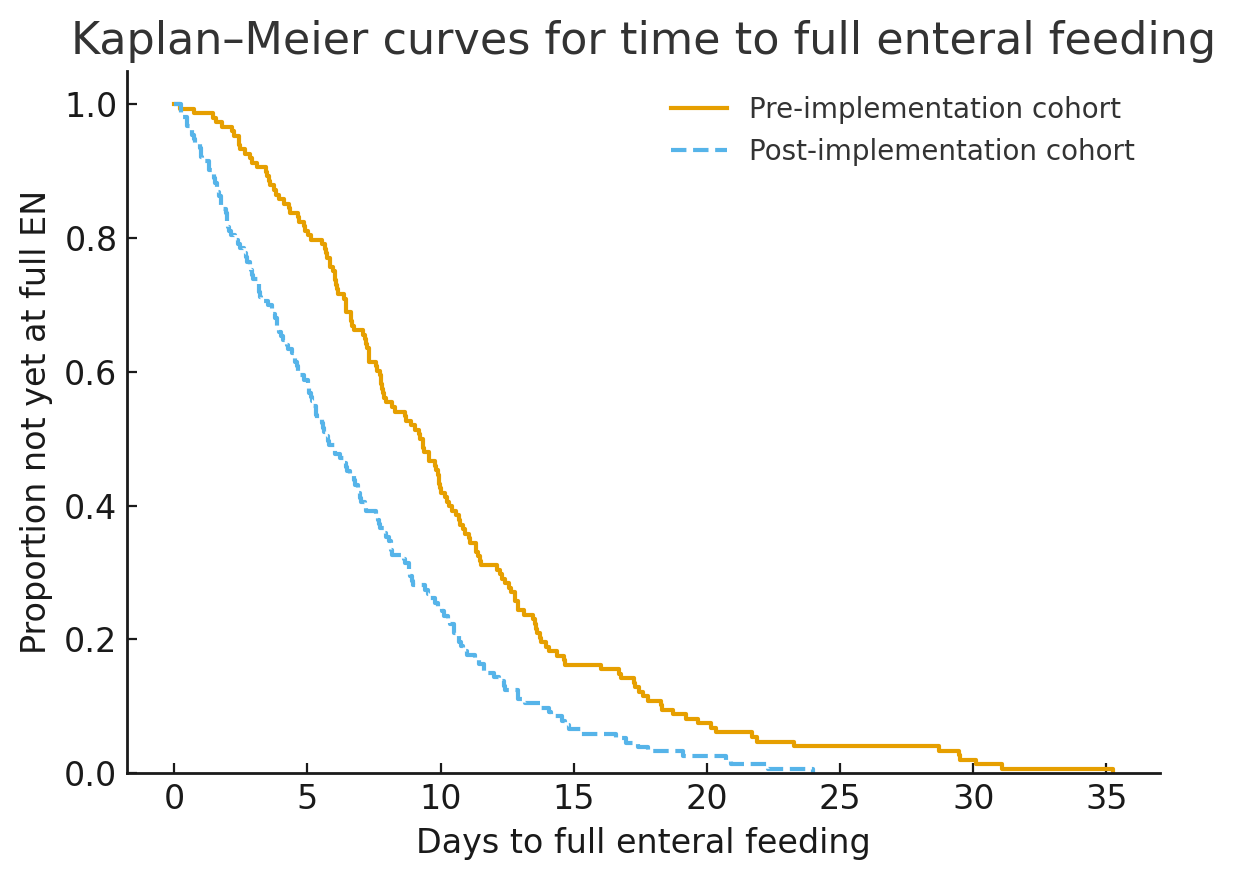


Supplementary Figure 2**.** Kaplan–Meier curves for time to full enteral feeding in the pre-implementation and post-implementation cohorts.

The post-implementation cohort reached full enteral feeding earlier than the pre-implementation cohort, as reflected by a left-shifted Kaplan–Meier curve (log-rank P < 0.05).


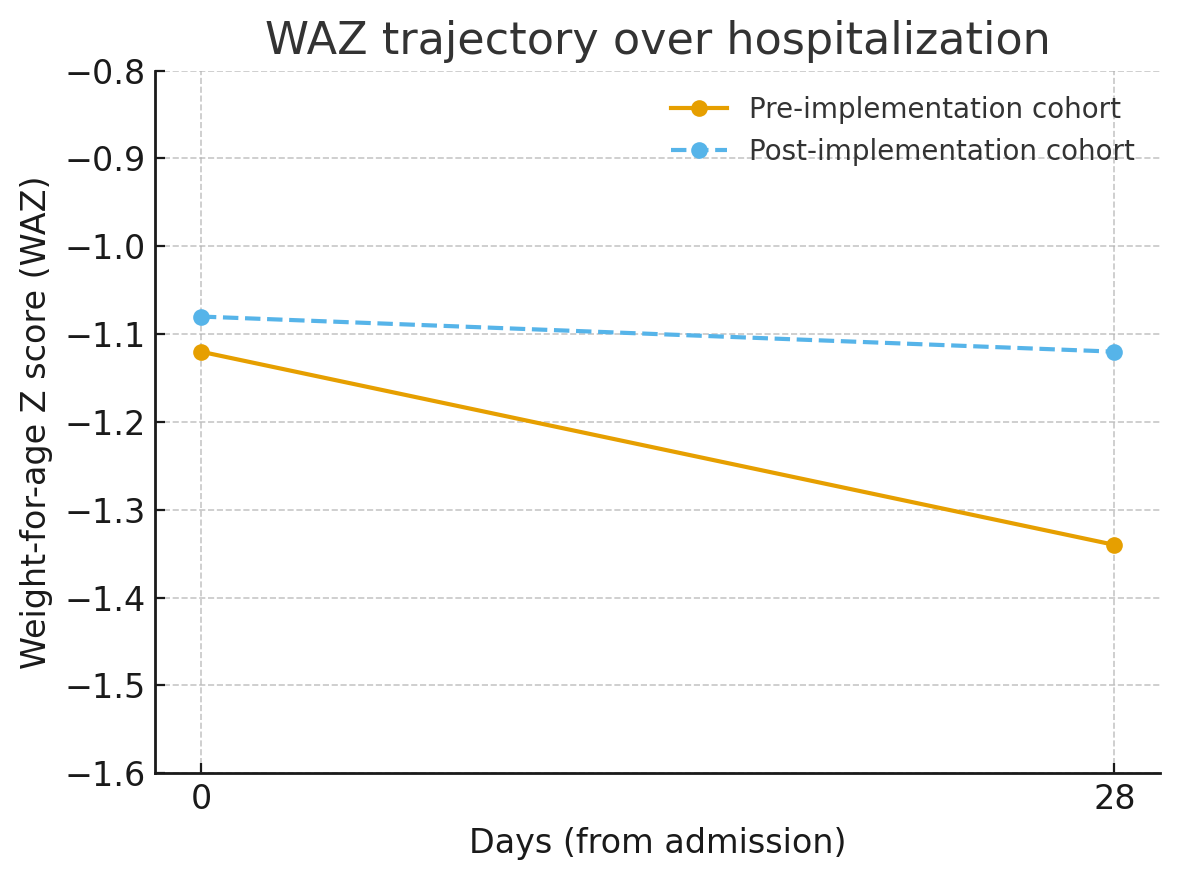


Supplementary Figure 3. Trajectory of weight-for-age Z score (WAZ) over hospitalization in pre- and post-implementation cohorts.

**Supplementary Table S1. Subgroup analysis in high-risk CHD strata (STAT 4–5 or single-ventricle physiology)**

| **Outcome** | **Pre-implementation (n = 62)** | **Post-implementation (n = 65)** | **Effect estimate** | **P value** |
| --- | --- | --- | --- | --- |
| Feeding intolerance, n (%) | 32 (51.61) | 22 (33.85) | aOR 0.54 (95% CI 0.31–0.96) | 0.034 |
| Time to full EN, median (IQR), days | 11.00 (8.00–14.00) | 8.00 (6.00–11.00) | HR 1.42 (95% CI 1.03–1.97) | 0.031 |

**Table Note**

Subgroup defined a priori as infants with STAT category 4–5 or single-ventricle repair.
Model adjusted for cardiopulmonary bypass time and peak lactate to reduce confounding by severity gradients.
aOR = adjusted odds ratio; HR = hazard ratio; CI = confidence interval.

**Supplementary Table S2. Enteral feeding initiation time subgroups (≤24 h vs >24 h)**

| **EN initiation timing subgroup** | **FI rate Pre (%)** | **FI rate Post (%)** | **Effect estimate** | **P value** |
| --- | --- | --- | --- | --- |
| ≤ 24 h post-op | 39.29 | 21.43 | aOR 0.47 (95% CI 0.24–0.94) | 0.032 |
| > 24 h post-op | 44.64 | 27.81 | aOR 0.61 (95% CI 0.35–1.08) | 0.089 |
| Time to full EN, ≤ 24 h, HR (95% CI) | — | — | HR 1.55 (95% CI 1.10–2.23) | 0.014 |
| Time to full EN, > 24 h, HR (95% CI) | — | — | HR 1.36 (95% CI 0.97–1.88) | 0.071 |

**Table Note**

Models stratified by predefined EN initiation thresholds (≤24 h vs >24 h).
No interaction term tested to avoid overfitting.
aOR adjusted for STAT category and ECMO status; HR adjusted for ICU length of stay.

**Supplementary Table S3. Sensitivity analyses for model robustness**

| **Sensitivity condition** | **Effect estimate** | **95% CI** | **P value** | **Interpretation** |
| --- | --- | --- | --- | --- |
| Excluding CICU stay > P95 (outliers) | aOR 0.50 | 0.30–0.83 | 0.006 | Association stable |
| Only infants completing DOL 28 follow-up | aOR 0.46 | 0.28–0.78 | 0.004 | No material deviation |
| Excluding preoperative EN exposure | aOR 0.52 | 0.31–0.87 | 0.012 | Direction unchanged |
| Adjusting additionally for vasoactive score | aOR 0.49 | 0.29–0.82 | 0.005 | Effect not attenuated |

**Table Note**

All sensitivity models retained the cohort term directionality without significant attenuation of effect size, supporting internal stability of observed estimates.
No imputation performed; complete-case framework applied.
